# Prediction of hub genes of Alzheimer’s disease using a protein interaction network and functional enrichment analysis

**DOI:** 10.5808/GI.2020.18.4.e39

**Published:** 2020-12-10

**Authors:** Jia Jin Wee, Suresh Kumar

**Affiliations:** Faculty of Health and Life Sciences, Management and Science University, 40100 Shah Alam, Malaysia

**Keywords:** Alzheimer’s disease, functional enrichment, hub genes, network analysis, protein-protein interaction

## Abstract

Alzheimer's disease (AD) is a chronic, progressive brain disorder that slowly destroys affected individuals’ memory and reasoning faculties, and consequently, their ability to perform the simplest tasks. This study investigated the hub genes of AD. Proteins interact with other proteins and non-protein molecules, and these interactions play an important role in understanding protein function. Computational methods are useful for understanding biological problems, in particular, network analyses of protein-protein interactions. Through a protein network analysis, we identified the following top 10 hub genes associated with AD: *PTGER3, C3AR1, NPY, ADCY2, CXCL12, CCR5, MTNR1A, CNR2, GRM2*, and *CXCL8*. Through gene enrichment, it was identified that most gene functions could be classified as integral to the plasma membrane, G-protein coupled receptor activity, and cell communication under gene ontology, as well as involvement in signal transduction pathways. Based on the convergent functional genomics ranking, the prioritized genes were *NPY, CXCL12, CCR5*, and *CNR2*.

## Introduction

As people become older, many parts of the body, including the brain, change. It is also normal for people to become forgetful, and age-associated memory impairment is considered to be part of the aging process. However, Alzheimer's disease (AD) is distinct from age-associated memory impairment, and instead is a type of dementia that causes problems with memory, thinking, and behavior. Hence, it is understandable that people, especially the elderly, are concerned about memory loss, as it is a symptom of AD. Dementia is known to be progressive, meaning that the condition becomes worse gradually. It is well known that AD has complicated and diverse pathogenic causes, including genetic, environmental, and immunological factors, as well as head trauma, depression, and hypertension. Moreover, genetic analyses have shown that human variations in AD can originate from several genes and their variants, which exert different biological functions in coordination to increase disease risk. AD typically occurs in elderly people (aged 65 years and above), while an uncommon variant known as early-onset AD comprises about 5% of AD cases [1]. As the name suggests, people with early-onset AD develop symptoms during their 40s and 50s, although the symptoms of both variants of AD are mostly the same.

According to the National Institute on Aging (https://www.nia.nih.gov/), some main characteristics of a brain with AD include amyloid plaques, neurofibrillary tangles, and chronic inflammation [2]. The amyloid plaques refer to beta-amyloid peptide (Aβ) which are the key components of amyloid plaques in brains affected by AD [3,4]. Abnormal levels and accumulation of Aβ form plaques that disrupt cell function. A similar process of unusual accumulations accounts for neurofibrillary tangles, which are driven by the intraneuronal accumulation of tau protein, which otherwise functions to stabilize microtubules, and cause AD [5,6]. Chronic inflammation is also linked to AD through the dysfunction of microglia in the central nervous system (CNS), which maintain homeostasis in the brain. The inability of microglia to function causes chronic inflammation.

Previous research has determined that carriers of the *APOE*-e4 risk gene have a higher risk of AD [7], and it is estimated that 40%‒65% of people with AD have that gene variant. Previous studies have mostly focused on gene regulatory networks in the late onset of AD [8] and the identification of active transcription factors by analyzing miRNA regulatory pathways [9]. To explore the molecular changes underlying AD, several genome-wide expression profiling experiments have been performed on the postmortem brain tissues of AD patients. However, the precise pathogenesis of AD remains unknown, and no effective treatment and prevention approaches are feasible. Apart from determining the pathways involved in AD pathogenesis, detailed analyses of possible candidate genes might lead to the identification of new strategies for predictive or diagnostic AD testing. In this study, we used a comprehensive database, DisGeNET, which includes information on all the genes related to AD to identify the hub genes involved in the disease. This study aimed to identify the hub genes involved in AD via protein-protein interactions.

## Methods

### Protein-protein interaction data collection

DisGeNET (https://www.disgenet.org/) [10] is a database containing information about human genes and variants. The data in DisGeNET are drawn from sources such as the scientific literature, animal models, and expertly curated repositories. Using the search tool in DisGeNET, the name of the disease (AD) was entered as a search query while choosing the disease search button. Then, the option “Summary of Gene-Disease Associations” was selected. A summary of all genes’ information, such as the HGNC gene symbol, UniProt ID, and protein class, was displayed. All of this information was downloaded in a Microsoft Excel file.

### Network construction and analysis

For this study, Cytoscape (https://cytoscape.org/) [11], an open-source software project, was used to construct the network by entering all the UniProt IDs [12] as search queries, while using the Search Tool for the Retrieval of Interacting Genes (STRING) for protein queries. STRING (https://string-db.org/) [13] is a database that contains information on known and predicted protein-protein interactions. Ambiguous terms were resolved by setting the confidence (score) cutoff to 0.4 and their maximum additional interactors to 0 to import the network.

### Hub gene identification

To determine the potential hub genes of AD, CytoHubba [14] was used to calculate the score for each node. This tool uses the maximal clique centrality (MCC) algorithm to show the top 10 ranked nodes, which could be the potential top 10 hub genes of AD. The top 10 genes were further analyzed for gene enrichment.

### Functional enrichment of hub genes

FunRich (http://www.funrich.org/) [15] was used for a functional enrichment and interaction network analysis of genes and proteins, using the gene ID of the top 10 genes. When applying the search, the analysis tab was selected and the bar graphs for four aspects (cellular component, molecular function, biological process, and biological pathway) were viewed, with the first six items of each category being shown on the chart.

### Gene prioritization using the AlzData database

AlzData is an integrated AD database that uses high-throughput omics data such as the results of genome-wide association studies (GWAS), whole-exome sequencing, transcriptome analysis, and proteomics to generate a prioritized gene list. We used this database to prioritize the top 10 hub genes identified.

## Results and Discussion

**Fig. 1.**
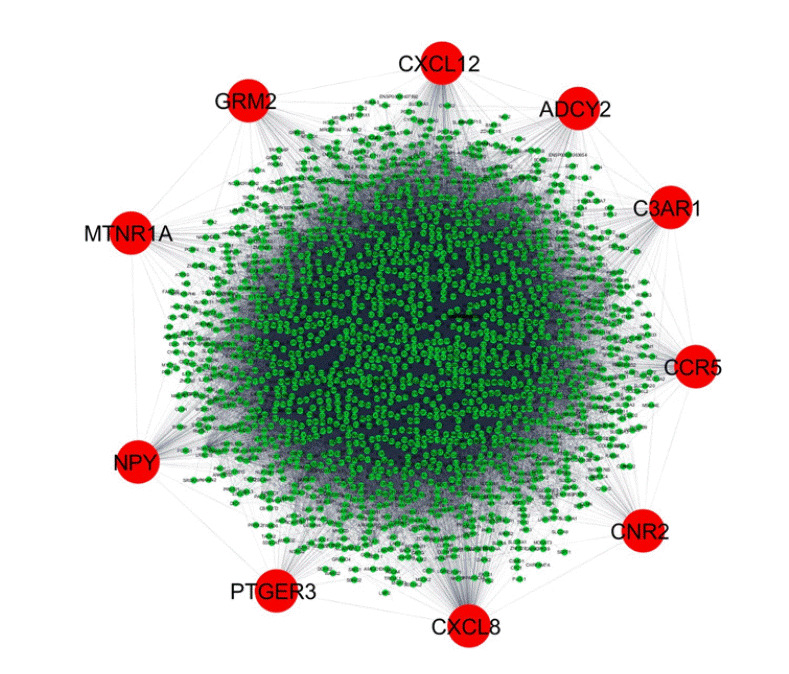
Overview of the protein-protein interaction network created using the STRING 10.0 database and Cytoscape software. The network has 1922 nodes and 57,617 edges. The analysis parameters are based on experiments, co-expression, and text mining with a 0.40 confidence score. The red circular nodes represent the hub genes interacting with other Alzheimer's disease‒associated genes, which are shown as small green circular nodes.

**Fig. 2.**
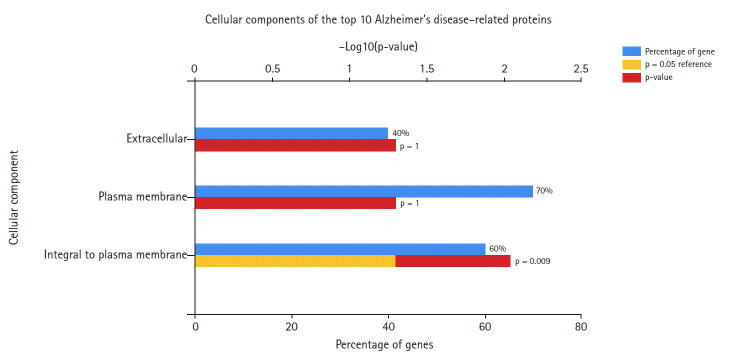
Cellular components of Alzheimer's disease hub genes.

**Fig. 3.**
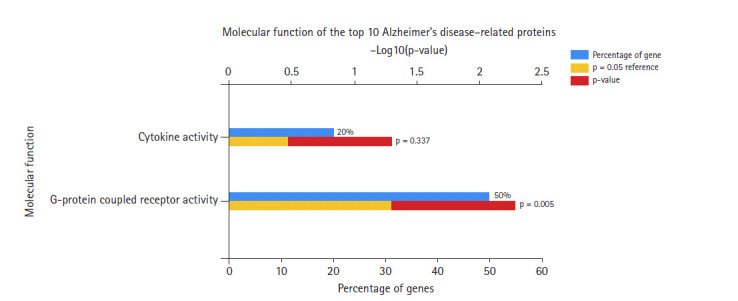
Molecular functions of Alzheimer's disease hub genes.

**Fig. 4.**
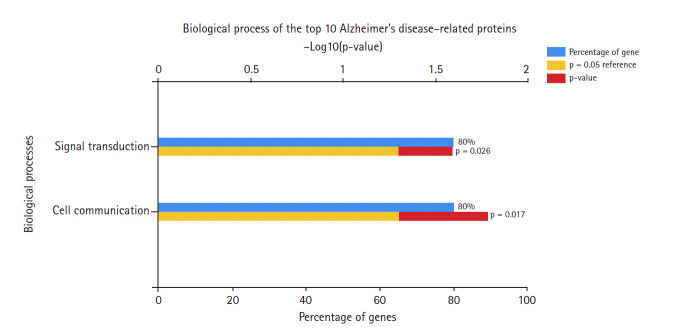
Biological processes of Alzheimer's disease hub genes.

**Fig. 5.**
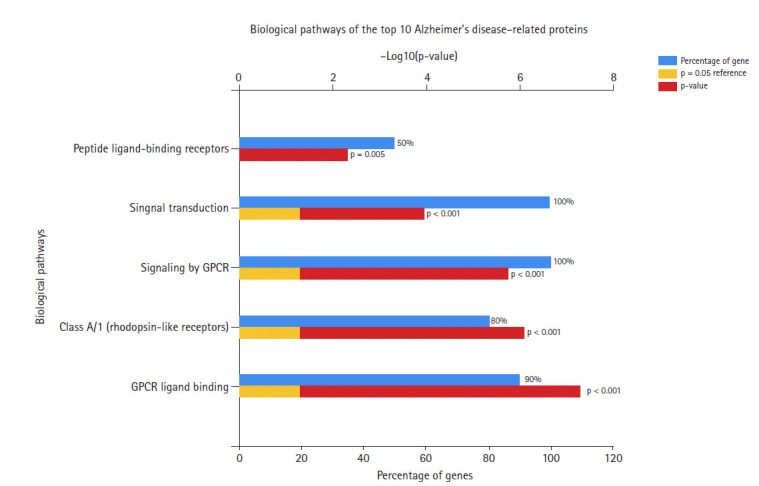
Biological pathways of Alzheimer's disease hub genes.

In the data downloaded from DisGeNET (accessed December 2019), there were 1981 genes involved in AD (Supplementary Table 1). A protein-protein interaction network was created by querying the STRING database for gene symbols with a confidence score of 0.4 to avoid false positives. The network of these genes had 1922 nodes and 57,617 edges, as shown in Fig. 1. Proteins with higher degrees in the network (hub genes) are more likely to be essential proteins. CytoHubba uses 11 methods to retrieve the top-ranked nodes. In CytoHubba, the MCC method captures more essential proteins in the top-ranked list in both high-degree and low-degree proteins. The top 10 nodes selected by MCC were all highly essential, meaning that these genes could be potential hub genes. These hub genes, by gene ID, were prostaglandin E receptor 3 *(PTGER3), C3AR1, NPY, ADCY2, CXCL12, CCR5, MTNR1A, CNR2, GRM2*, and *CXCL8*. The results of the gene enrichment analysis for cellular components (Fig. 2), molecular function (Fig. 3), biological processes (Fig. 4), and biological pathways (Fig. 5) were displayed in the bar graphs.

Fig. 2 shows that the highest percentage of these genes was found at the plasma membrane, and including genes integral to the plasma membrane. The extracellular component accounted for the lowest percentage of AD hub genes.

Regarding the molecular function of the AD hub genes (Fig. 3), most of the genes were related to G-protein coupled receptor (GPCR) activity, and were rarely involved in other activities such as cytokine receptor, chemokine, and adenylate cyclase activity.

In terms of biological processes (Fig. 4), most of these hub genes were involved in signal transduction and cell communication processes, rather than other processes such as the immune response or other unknown processes.

Fig. 5 shows the biological pathways of the hub genes. Most of these genes are involved in signal transduction and signaling by GPCR, as well as the class A/1 (rhodopsin-like receptors) pathway, peptide ligand-binding receptors, and chemokine receptors.

PTGER3 and prostaglandin E2 are derived from the metabolism of arachidonic acid by cyclooxygenases in the cyclooxygenase pathway. This protein is the main neuroinflammatory molecule [16], and its receptor EP3 subtype is highly expressed in the brain. Studies have shown that activation of the EP3 receptor can reduce or suppress cyclic adenosine monophosphate (cAMP) formation [17]. This affects the microglia, thereby causing many brain diseases including AD.

*C3AR1*, or complement C3a receptor 1, is another gene involved in AD. C3a is an anaphylatoxin released during activation of the complement system. The *C3AR1* gene encodes the orphan GPCR for C3a. It also has essential functions in the immune response and host defense. A study has shown the importance of activation of the C3-C3aR network in mediating neuroinflammation and tau pathology [6]. The tau protein is a key protein that has been implicated in many neurodegenerative diseases such as AD and Parkinson disease.

A widely expressed gene in the CNS, *NPY* encodes neuropeptide Y. Neuropeptides are signaling molecules that influence brain activity in specific ways. They are involved in the pathophysiology of AD, and those with AD were found to have notably lower plasma levels of neuropeptide Y than healthy individuals [18]. *ADCY2* encodes the protein adenylate cyclase 2. This, according to the NCBI, is a membrane-associated enzyme that catalyzes the formation of cAMP, which is a messenger for intracellular signal induction. A study reported that in patients with AD, cAMP activity was higher in cerebral microvessels than in healthy individuals [19].

The *CXCL12* gene encodes the chemokine protein named C-X-C motif chemokine 12, which is a member of the intracrine family of stromal cell-derived chemokines and is involved in the CXCL12-CXCL4 pathway. This molecule regulates neuronal excitability and synaptic transmission [20]. A test conducted using a mouse model [21], proved that patients with AD had reduced amounts of CXCL12. This, in turn, is linked to the fact that these patients have impaired learning and memory, which is also a symptom of AD. The C-C chemokine receptor family is a main inflammatory receptor family that has been found to be involved in AD [22]. CCR5 or C-C motif chemokine receptor 5 is one such receptor. Many studies have demonstrated upregulation of this chemokine receptor in patients with AD, and it has been reported to recruit microglia and to cause accumulations of microglia in senile plaques [23], thereby accelerating AD development [22].

*MTNR1A* encodes melatonin receptor 1A. Melatonin is a hormone that is released in the pineal gland and regulates the sleep-wake cycle. Furthermore, melatonin has been reported to have neuroprotective and anti-amyloidogenic effects, as it reduced Aβ production in multiple neuronal cell lines [3]. As people age, the secretion of this hormone decreases, and low levels of melatonin contribute to aging.

The endocannabinoid system (ECS) comprises endocannabinoids, which are endogenous lipid-based retrograde neurotransmitters that bind to cannabinoid receptors. The ECS is involved in regulating physiological and cognitive processes, including include memory. One of the key receptors, cannabinoid receptor 2, is encoded by *CNR2*. Imbalances in the ECS, including elevated expression of glial cannabinoid receptor 2, in AD models suggest its potential role in inflammatory and neuroprotective processes [24].

*GRM2*, which stands for glutamate metabotropic receptor 2, encodes a protein named metabotropic glutamate receptor 2 (mGluR2). Glutamate is the main excitatory neurotransmitter in the CNS, and mGluR2 modulates rapid synaptic transmission in the CNS via controlled release of the excitatory amino acid glutamate [25]. Altered glutamatergic synaptic transmission is a key event in the development of AD.

*CXCL8* encodes C-X-C motif chemokine ligand 8, which is a member of the CXC chemokine family that is also known as interleukin 8 (IL-8). Inflammatory processes have been found to be involved in neurodegenerative disorders such as AD, and the involvement of chemokines such as IL-8 has been reported to be involved in these inflammatory processes [26].

We used the convergent functional genomics (CFG) ranking for target genes available in the AlzData database. AlzData (http://www.alzdata.org/) integrates five lines of evidence associated with AD. One CFG point is assigned for each piece of evidence (e.g., expression of the target gene is regulated by AD genetic variants in GWAS; the target gene has significant physical interactions with *APP, PSEN1, PSEN2, APOE*, or *MAPT*; the target gene is differentially expressed in AD mouse models before AD pathology emergence; the target gene expression is correlated with AD pathology in Aβ-line AD mouse models and tau line AD mouse models). The number of CFG points ranges from 0 to 5. According to the CFG ranking, the top four genes (with 4 points each) were *NPY, CXCL12, CCR5*, and *CNR2*, and the genes in second place (with 2 points each) were *PTGER3, MTNR1A*, and *GRM2* (Table 1). Several approaches have been used in previous research to identify potential target genes, such as *ZFHX3, ERBB2, ERBB4, OCT3, MIF, CDK13*, and *GPI* [27-33]. The most recent gene expression analysis conducted by Yan et al. [34] identified the following hub genes: *CDC42, VEGFA, BDNF, PDYN, CALB, TH, CACNA1A, OXT, CD44*, and *TAC1*. The genes identified by Wu et al. [35] were *ITGB5, RPH3A, GNAS*, and *THY1*. Thus, the present study found a few previously unreported novel hub genes, as follows: *PTGER3, C3AR1, NPY, ADCY2, CXCL12, CCR5, MTNR1A, CNR2, GRM2*, and *CXCL8*. The priority genes were identified as *NPY, CXCL12, CCR5*, and *CNR2* based on the CFG ranking.

Protein-protein interactions are important for understanding protein function and behavior. In this study, we identified 10 hub genes of AD. The results show that most of these genes encode receptor proteins that are involved in biological pathways in the plasma membrane. The hub genes identified through a network analysis can be used as targets to suppress AD in patients. Our analysis can shed some light on a deeper understanding of the fundamental molecular pathways and key molecular players of AD and offers a new point of view for researchers studying the causes of AD.

## Figures and Tables

**Table 1.** CFG ranking of top the 10 hub genes of AD

Gene	eQTL	GWAS	PPI	Early_DEG	Pathology cor (Aβ)	Pathology cor (tau)	CFG
*PTGER3*	1	0	APP, PSEN1, APOE	NA	NA	NA	2
*C3AR1*	0	0	APP, APOE	NA	NA	NA	1
*NPY*	NA	1	APP	Yes	‒0.374*	0.166 ns	4
*ADCY2*	NA	0	APP	NA	‒0.082 ns	‒0.481 ns	1
*CXCL12*	1	0	APP, PSEN1, MAPT, APOE	Yes	0.432**	‒0.069 ns	4
*CCR5*	1	0	APP	Yes	0.769***	0.616*	4
*MTNR1A*	1	0	APP, PSEN1, APOE	NA	NA	NA	2
*CNR2*	1	0	APP, PSEN1, APOE	Yes	0.854***	0.750**	4
*GRM2*	1	0	APP	NA	NA	NA	2
*CXCL8*	NA	0	NA	NA	NA	NA	0

CFG, convergent functional genomics; AD, Alzheimer's disease; GWAS, genome-wide association studies; PPI, protein protein interaction; DEG, differentially expressed gene; Aβ, beta-amyloids; NA, not available. eQTL: expression of target gene is regulated by AD genetic variants (genetic variants: IGAP GWAS P < 1E-3; eQTL: p < 1E-3); GWAS: IGAP p < 1E-3; PPI: target gene has significant physical interaction with APP, PSEN1, PSEN2, APOE, or MAPT (p < 0.05); Early_DEG: target gene is differentially expressed in AD mouse models before AD pathology emergence; Pathology cor (Aβ): correlation of target gene expression with AD pathology in Aβ-line AD mouse models (r, p; ns, p > 0.05; *p <0.05, **p < 0.01, ***p < 0.001); Pathology cor (tau): correlation of target gene expression with AD pathology in tau line AD mouse models (r, p; ns, p > 0.05, *p < 0.05, **p < 0.01, ***p < 0.001); CFG: total CFG score of a target gene, 1 CFG point is assigned if any of the above evidence is significant, the total of CFG points ranges from 0 to 5.

## Supplementary Materials

Supplementary Table 1. List of 1,981 genes associated with Alzheimer's disease retrieved from Disgenet database
