# Supplementary material for: Prediction of hub genes of Alzheimer’s disease using a protein interaction network and functional enrichment analysis
Source: Genomics Inform. 2020 Dec 10;18(4):e39. doi: 10.5808/GI.2020.18.4.e39 (PMC7808865; doi:10.5808/GI.2020.18.4.e39)
Supplement: Supplementary Table 1. — List of 1,981 genes associated with Alzheimer's disease retrieved from Disgenet database [file gi-2020-18-4-e39-suppl.pdf]

Supplementary Table 1. List of 1,981 genes associated with Alzheimer disease retrieved from Disgenet database

List of genes associated with Alzheimer disease
APP,APOE,PSEN1,SORL1,BCL2,BDNF,ACE,GSK3B,IL1B,INSR,LEP,PLAU,IGF2,NPY,IGF1R,INS,BAX,CLU,PSEN2,PICALM,ABCA7,CD2AP,TREM2,CR1,VSNL1,PCDH11X,MS4A4A,A2M,ACHE,BIN1,BCHE,CALM1,CASP3,CD33,CHRNA7,CST3,CYP2D6,DHCR24,DPYSL2,EPHA1,ESR1,HFE,HMOX1,IDE,IGF1,IL6,MAPT,MPO,MTHFR,NOS3,PPARG,PRNP,RELN,TNF,VEGFA,CYP46A1,NCSTN,BACE1,MIR146A,BLMH,MAOB,SOD2,TF,TFAM,APBB2,CHRNB2,SLC30A6,EIF2S1,ATP5F1A,CRH,ENO1,F2,IGF2R,PAXIP1,ARC,HLA-DRB5,TPI1,SLC30A4,ABI3,AGER,AMFR,CHAT,TPP1,LRP1,MME,NGF,NGFR,PIN1,PLCG2,PYY,PTGS2,S100B,SLC2A4,SOD1,GAPDHS,NFE2L2,NOS2,MAP2,NTRK2,IRS1,APLP2,PTGS1,PPARGC1A,NGB,CAV1,MAPK14,CTNNB1,CCR5,HSPB1,HSPD1,MFN2,CIB1,ATP7A,GSR,HSF1,IKBKB,INS-IGF2,MT2A,SERPINF1,GAB2,TOMM40,PLCB1,SQSTM1,FERMT2,EXOC3L2,MS4A4E,NFIC,CELF1,ZCWPW1,FRMD4A,MS4A6A,PTK2B,IL6R,INPP5D,TRIP4,BCL3,NR3C2,SUCLG2,SPON1,CELF2,MTHFD1L,DCHS2,CASS4,EXOC4,MEGF10,PLXNA4,SLC24A4,GLIS3,SERPINA3,ABCA1,ADAM10,AP2A2,ALOX5,APBB1,APOA1,DST,RUNX1T1,CDK5,CDR1,CETP,CHRNA4,CLPTM1,ACKR2,CP,CRP,CSF2,CTNNA2,CTSB,CTSD,DMXL1,DLG4,RCAN1,DYRK1A,ENO2,ESR2,MTOR,GABRG3,GAPDH,GATA1,GFAP,GRN,GRIN2B,HSD17B10,HTR2A,IL1A,IL10,KCNN2,LAMC2,RPSA,LDLR,LTBP2,BCAM,AFF1,MMP9,MOBP,COX2,RNR2,NTF3,ABCB1,PON1,PTPA,MAPK1,PTPRA,PTPRG,NECTIN2,MOK,CCL2,ST6GAL1,SLC1A2,SLC6A4,SNCA,SST,SYP,TGFB1,TP53,TTR,UBB,UCHL1,AGPS,CRADD,SUCLA2,CDK5R1,CACNA1G,CCRL2,SLC16A7,LRAT,ITM2B,SLC4A8,ST18,MVP,DNM1L,GPC6,FARP1,MYO16,ZNF292,FBXL7,PRRC2C,CLEC16A,WWC1,ACSL6,SIRT1,NCS1,TARDBP,SMUG1,BACE2,TRPC4AP,PDE7B,TNRC6A,BZW2,PARVB,IL19,UBQLN1,RMDN1,RAPGEF6,BCAS3,CDKAL1,RMDN3,STK32B,SYBU,EDEM2,FMN2,CDC42SE2,ARHGAP20,SH3RF1,VAT1L,CSMD1,PPP1R3B,ANKRD55,CLMN,SP6,SPPL2A,FNIP1,PALM2,OSBPL6,LRRK2,ANO4,NDUFAF6,RMDN2,NKAIN2,BMPER,PLPP4,C9orf72,CCDC83,STH,CALHM1,PPP1R37,NKPD1,EPHA1-AS1,LUZP2,TGM6,MCIDAS,SIMC1,SCIMP,LINC01567,PALM2-AKAP2,LINC01184,MEIKIN,SLC8A1-AS1,ARL17B,TSPOAP1-

AS1,LINC01725,MEF2C-AS1,LINC00972,ABCA2,APOD,FAS,BRCA2,CALM2,CALM3,COMT,CYP19A1,EPHB2,FANCD2,IFNG,LPL,NFKB1,NPC1,OGDH,SLC6A3,VLDLR,ALB,APOC1,CD36,CD40,DLST,EEF1A2,NR3C1,CFH,NEFL,PLG,MAPK8,EIF2AK2,SNAP25,TLR4,TSHZ1,YWHAQ,COL25A1,CAPN1,CDK1,CREB1,DAPK1,GAP43,IAPP,MT3,PIK3CA,PIK3CG,PLA2G1B,TFCP2,YWHAZ,RTN3,HPGDS,SORCS1,SERPINA13P,MIR132,ACAT1,PARP1,APBA1,ATP7B,CASP6,CASR,CD44,CETN1,COX8A,CREBBP,FYN,GSN,IL1RN,IL12A,IL18,ITGAM,KCNA3,MAOA,NOS1,OLR1,SERPINE1,PIK3CB,PIK3CD,PKLR,POLD1,RAB5A,SORT1,SOAT1,TFF1,THY1,DENR,SDS,PLD3,QPCT,SDF4,PLB1,FSIP1,TAS2R62P,TAS2R64P,ABCA4,ACTB,AGT,ALDH2,ANXA1,APLP1,BLVRA,C4A,C4B,DDR1,CALB1,CAST,CD40LG,CNR2,CRMP1,DBN1,DECR1,ECE1,ELANE,FCN2,GCHFR,GRM5,GTF2H1,GZMB,HMGCR,HSPA4,IGFALS,IGFBP3,LCN2,LPA,MMP3,MNAT1,COX1,PAWR,SERPINA1,PLA2G2A,PLA2G4A,PLTP,MAPK3,ROS1,STAT3,TH,TLR2,TNFRSF1A,UTRN,VDAC1,VDR,UNC5C,HDAC6,KHDRBS1,TPPP,DKK1,SIRT3,DAPK2,NUP62,CTNNA3,GAL,DCTN4,GSAP,TMEM106B,SEMA6A,PINK1,RTN4R,CDCA5,SLCO6A1,C4B_2,BACE1-AS,UPK3B,ADORA2A,APOB,AQP4,AR,ATF4,TSPO,C3,CD59,CD68,CRYAB,DNM2,EGR1,EIF4E,MARK2,ETS2,FGF1,GABPA,GCG,GDNF,GH1,GRIA1,GRIA2,GRM2,HCRT,NRG1,HMGA1,HRES1,HSD11B1,HSPA1A,HSPA1B,HSPA5,HSP90AA1,HSPG2,IL4,CXCL8,IL13,KLC1,LRP2,MGAT3,NTRK1,NTSR1,OGG1,PON2,PPARA,PTGDS,PTPRC,RAC1,OPN1LW,REN,S100A9,SNCB,SP1,TBP,PRDX2,TGM2,TNFRSF1B,VCAM1,VIM,PLA2G6,PDE5A,BECN1,XPR1,ABCG2,GSTO1,ABCG1,TUBA1B,KAT5,OGA,SIRT2,TIPARP,SGSM3,TLR9,TMED9,VPS35,PAG1,PSENEN,PCBP4,MMEL1,TREML2,APH1B,DCTN5,TTBK1,ATCAY,LRRTM3,GSTK1,POTEF,PGR-AS1,GRK2,AGTR2,AIF1,ALOX15,ANK1,APCS,APOA4,APRT,AQP1,ARNTL,ARR3,STS,ATM,BRCA1,BSG,C2,C5AR1,CAMK2A,CASP1,CBS,CDK4,CEBPD,CHI3L1,CHRM2,CNTF,CPOX,CRHR1,CUX1,CX3CR1,CXADR,CYP2B6,CYP3A4,DAB1,DBH,DCX,DNM1,DRD4,ELAVL2,ELK3,ERBB4,F2R,FDPS,FGF2,FKBP4,FPR2,GLUL,GRIN2A,GSTM1,GSTM3,GSTP1,HTT,UBE2K,HLA-A,HLA-DRB1,HNRNPA1,HSPA9,HSPB2,HTR4,HTR6,ICAM1,IL2,ITGAX,KNG1,LHCGR,LIPA,MBP,MELTF,MMP2,NEDD9,NFATC2,NFIA,NFIB,NFIX,NOTCH1,NOTCH3,PRKN,PCK1,PCNA,PECAM1,PHF1,PPY,PRKAA1,PRKAA2,PRKAB1,PRKAR1A,MAP2K1,KLK6,PTBP1,RPS6KB1,S100A1,CCL5,CX3CL1,SET,SLC6A2,SNCG,SPG7,SRPK2,

ADAM17,TAP2,TAT,TP73,TSC2,TYROBP,NR1H2,XBP1,XPNPEP1,YY1,PLA2G7,NR4A3,PABPC4,NR1I2,SYNJ1,F2RL3,CH25H,PPIG,KL,CLOCK,NR1I3,RANBP9,ABCB6,ABCC9,TRIM13,SIGMAR1,AKR1A1,C1D,ADAP1,CHEK2,NLRP1,SPEN,GGA3,DNMBP,CRTC1,PYCARD,KCNIP3,APH1A,GOLM1,NME8,CALML5,SAMD9,LRRC4,FTO,SLC52A2,NLRP3,ESCO1,GSTO2,PWAR1,C1orf52,MS4A6E,PWAR4,MIR107,MIR29A,MIR342,CXADRP1,HNRNPA1P10,C20orf181,AD11,LINC02210-CRHR1,NAT2,ABO,ACO1,ADRB1,ADRB2,AGTR1,AKT1,ANXA5,ANXA6,APC,APOC2,ARRB2,BACH1,BAG1,CFB,BMP4,SERPING1,CALR,CASP8,CD14,CD69,CDH1,CDKN1A,CDKN1B,CDKN2A,CEBPB,CES1,AKR1C4,CHEK1,CHM,CHRNA3,CKB,CNR1,COL3A1,CRK,CSF1,CSNK1D,CTGF,CTNND2,CYP2C9,GADD45A,DDIT3,NQO1,DLD,DPYSL3,DRD1,E2F1,EEF2,EGFR,F13A1,FAAH,FKBP5,FLG,FLNA,FN1,FOS,FXN,GAD1,GBA,GIP,GJA1,GLB1,GLO1,GLP1R,GPC1,GRB2,GRIN1,GSTT1,HCLS1,HDAC2,HHEX,HMOX2,HP,HPS1,HSPA8,HTR1A,HTR2C,IL2RB,IL6ST,IL9,CXCR2,IL9R,IL17A,ITPR3,JAK2,LAMP1,STMN1,LIF,LMNB1,LNPEP,LRP6,MARK1,CD46,MEF2C,MEOX2,MIF,MYC,NUBP1,NFATC4,NM,NOTCH4,NPPA,SLC11A2,ODC1,OPRD1,P2RX7,P2RY2,P4HB,PRDX1,PAK1,PDE4A,PDK1,PLD2,SERPINF2,POLB,POMC,PON3,POU2F1,PPID,MAPK10,MAP2K2,HTRA1,PTGER2,RAB3A,RAB4A,RENBP,REST,RPS6,RXRA,RYR3,SCD,CCL3,CCL11,CXCL12,SRSF2,SHBG,ST8SIA1,SLC2A1,SYN1,TGFB3,TGFBR2,TGM1,TIMP1,TIMP2,HSP90B2P,TTPA,TXNRD1,SUMO1,UGCG,USF1,VCP,XK,SLC30A1,SLC30A3,CXCR4,CP20,AIMP2,FZD4,OGT,MADD,KHSRP,TP63,HSD17B6,TRADD,TNFSF10,HAP1,MSC,COX5A,GRAP2,MED23,EIF2AK3,MAPK8IP1,PTGES,APBA3,VPS26A,PRDX6,KLK4,RIMS2,HDAC4,NR1H3,LRPPRC,OPTN,AKAP9,FLOT1,SPAG11B,OLFM1,GPNMB,AHSA1,NES,PLK2,PSIP1,SHANK2,SYNM,ADNP,SRRM2,LPAR3,PPIL2,CIZ1,RNF19A,APPL1,POLDIP2,GGA1,SIGLEC7,DISC1,RBMS3,PCSK1N,HTRA2,DLL1,EEF2K,ERVW-1,PLA2G3,HSPA14,PRRX2,HSD17B7,WWOX,DDIT4,PNO1,SLC17A7,MCOLN1,NDRG2,LGR6,ABCG4,WNK1,DHX40,EHMT1,COASY,COL18A1,BHLHB9,HM13,ARHGAP24,PPP1R1B,SYVN1,EBPL,UBASH3B,NAV3,CHRFAM7A,IL33,MTG1,PRRT2,MRGPRX3,MRGPRX4,HSPB6,RBM45,GPR151,SLC2A14,RBFOX3,PDIK1L,GPBAR1,DPPA2,OXER1,GPRC6A,MRGPRX1,MOGAT3,AGRN,ARMS2,MIR206,MIR212,MIR29B1,MIR29B2,MIR29C,EIF2AK4,VN1R17P,GPR166P,MIR424,LOC643387,SPAG11A,ZGLP1,CASP12,CBSL,AAVS1,ABL1,ACADVL,ACO2,ADARB1,ADCY2,ADCYAP1,ADD3,ADM,ADRA2A,ACAN,AHR,AHSG,ALOX12,AMD1,AMD1P2,AMPH,ANG,ANGPT

2,ANK3,SLC25A4,AOC2,APBA2,APEH,BIRC3,XIAP,BIRC5,APOA2,APOC3,KLK3,FASLG,ARG2,RHOA,SERPINC1,ZFHX3,ATP12A,ATP4A,ATP5MC1,ATP5MC2,ATP5PF,AVP,AZU1,CCND1,BCL2L2,BCS1L,BCYRN1,BDKRB2,BID,BLM,BMP6,BRS3,BST1,C3AR1,C5,C9,C21orf2,CA2,SLC25A20,CALB2,CALCA,CALCR,CAMK4,CAPG,CAPN2,CASP4,CASP7,CASP9,CAT,CCK,CCNC,CD5L,CD8A,MS4A1,MS4A3,CD80,CD86,CD34,CD38,CD47,CD53,CD74,CDA,CDC25B,CDC42,CDH2,CDK9,CEACAM5,CECR,CFL2,CFTR,CHGA,CHGB,CHRM1,CISH,CLCN3,CLK1,CLK2,CCR6,CMKLR1,ABCC2,CNP,COL11A2,SLC31A1,COX10,COX15,CPB1,CPE,CPN1,ATF2,CSF1R,CSF3,CSNK2A1,VCAN,CST5,CST6,CTF1,CTSK,CTSS,CTSZ,CYBB,CYP1A2,CYP2C19,CYP11B1,CYP11B2,CYP17A1,CYP27A1,DAXX,DCN,AKR1C2,Sep-01,DIO1,DIO2,DLG1,DLG3,DMD,DMRT1,DNMT1,DOCK2,DPP4,DRD2,DRD3,ATN1,DSG1,DSG2,DUSP6,DVL1,EDN1,EGF,EGR2,EIF2S3,EIF4A1,EIF4A2,EIF4EBP1,EIF4G2,ELAVL4,EMP1,CTTN,ENPEP,EP300,EPHA4,EPHB1,EPO,EPOR,ERBB2,ERG,EZH2,F2RL1,F7,F11,F13B,FABP3,BPTF,FANCG,FASN,FAT1,MS4A2,FCGR3A,FCGR3B,FES,FGF14,FGFR3,FHL2,FKBP1A,FOXM1,FOXO1,FOXO3,FLNB,FLT4,FMR1,FOLH1,FOSB,FRK,FSHR,GABBR1,GART,GFER,GFRA1,GFRA3,GGT1,GJA8,GLI1,GMFB,GNAI1,GNB3,GOLGA2,GPI,UTS2R,GRK5,GPT,GRIA3,GRIK4,CXCL1,GSM1,GYPA,GYPB,GYPE,HAGH,HADH,HARS,HBG2,HCRTR2,HDAC1,HDC,HGF,HIF1A,HIP1,HK1,HLA-B,HLA-DRA,HLA-DRB4,HLA-G,HMGB1,HMGCS2,FOXA1,FOXA2,HNF4A,HNRNPA2B1,HPCA,HSD17B1,HSD17B4,IDUA,IFI27,IFIT3,IFNA1,IFNA13,IFNB1,IGFBP1,IGFBP5,IGFBP7,IGL,IL1R1,IL1RAP,IL12B,ILK,IDO1,ING1,INPPL1,IREB2,IRF6,IRF7,ITGAV,ITGB1,ITGB3,ITPR1,ITPR2,JUN,JUNB,JUND,KCNB1,KCNC4,KCNMA1,KCNQ1,KLRC1,KIF11,KRT14,LAMC1,LAMP2,LBP,LDHA,LIFR,LIMS1,LIPC,LMNA,LOX,LOXL1,LRPAP1,LYZ,MARCKS,SMAD2,MAP1A,MAP1B,MAT1A,MAT2A,MAZ,MBL2,MDK,MDM2,MEF2A,MEF2D,MEFV,MET,MFGE8,MICE,ATXN3,MLLT3,MMP1,MMP14,MOG,CD200,MPI,MPZ,MRE11,ABCC1,MSD,MSI1,MSRA,COX3,MTHFD1,ND1,ND4,MTNR1A,MTR,MTRR,MUT,MX1,MYCL,MYD88,MYH6,MYO6,NACA,NCAM2,NDUFA5,NDUFA6,NDUFB8,Sep-02,NEFM,NEFH,NEU1,NINJ2,NME1,NME2,NQO2,PNP,NPHP1,NPTX1,NRDC,NRGN,YBX1,NTS,NUP98,NR4A2,OMP,OSM,OTC,OXA1L,OXT,P2RY1,P2RY4,P2RY6,PEBP1,PAX6,PC,PCP4,CDK18,PDE7A,PDGFB,PDGFRB,ENPP2,PDYN,PENK,PER1,PFKFB3,SERPINE2,SERPINI1,PIK3C3,PLAT,PLAUR,PLCL1,PLD1,PLEK,PLK1,P

ML,PMM2,PRRX1,POLG,POU3F4,POU5F1,PPARD,PPIA,PPIB,PPP1CA,PPP1R1A
,PPP1R10,PPP2CA,PPP2R2B,PPP3CA,PPP3R1,PPT1,PRB1,PRKACA,PRKACB,PR
KAR1B,PRKCB,PRKCE,PRL,PROS2P,PRSS3,MASP1,PSD,PSMB6,PSMB9,PSMD2,P
SMD9,PTCH1,PTEN,PTGDR,PTGER1,PTGER3,PTGER4,PTPN1,PURA,PVALB,PZP
,RAB6A,RAD23B,RAF1,RAN,RB1,RBBP6,RBP4,REG1A,RELA,RET,RFC1,RGS2,RGS
4,RHD,ROCK1,RPL13,RPS3A,RPS6KB2,RPS23,RPS27,RRAS,RYR2,S100A6,S100A8,
S100A12,ATXN1,ATXN2,SCP2,CCL8,CCL19,CCL21,SDC2,SEL1L,MAP2K4,SFPQ,S
RSF3,SRSF5,TRA2B,SGCA,SGSH,ITSN1,SH3GL2,SIM2,SKIL,SLC1A1,SLC1A3,SLC2
A3,SLC8A1,SLC8A3,SLC18A1,SLC18A2,SLIT3,SMPD1,SMPD2,SOS2,SOX2,SP4,SPA
ST,SPTBN1,SREBF2,SRF,SSTR2,ST2,STAT1,STC1,STXBP1,SULT2A1,VAMP1,SYN2,
SYT1,TACR2,TACR3,TRBV20OR92,TDO2,TERT,TFDP1,TFF3,TFRC,TGFB2,THAS,
THBS4,THOP1,THRA,TIA1,TIMP3,TIMP4,TLE1,TSPAN7,CLDN5,TNFAIP1,TNNI3,T
P53BP2,TPH1,TPT1,TRAF2,TRPC6,TRPM2,TSHR,TSPY1,TTK,TWIST1,TYK2,TYRO
3,TYRP1,UBE3A,SLC35A2,UGT1A,UNG,UQCRC1,USF2,VIP,BEST1,VWF,WARS,W
AS,WEE1,WNT1,WNT5A,WNT2B,WT1,XBP1P1,XRCC1,MZF1,RNF112,ZNF224,ZNF
236,MAP3K12,LRP8,FZD5,RAB7A,REEP5,DDX39B,TFEB,TFPI2,ARHGEF5,PSCA,
NUP214,AD5,AAAS,PABPN1,ANP32A,CDR3,SLC14A2,CHAF1B,USP9X,DYSF,BAP
1,EOMES,DHX16,FKBP6,IRS4,PKP4,PIK3R3,LMO4,MAPKAPK5,PRKRA,STK16,US
O1,PDE8B,AOC3,NUMB,SOCS1,IRS2,TNK1,RIPK1,ADAM9,RAB11A,TMEM11,SOC
S2,HDAC3,PROM1,HCAR3,ALDH1A2,PER3,PER2,ARHGEF7,NAE1,EIF2S2,WASF1
,CDK5R2,HSPB3,KALRN,SOCS3,BRSK2,NAT8,SPAG9,ARTN,CLDN1,CBFA2T2,HG
S,IL1RL1,SLC33A1,ZMYM3,TIAF1,RAB11B,NOG,MAPKAPK2,SOCS6,CD83,TAOK2
,SLIT2,CYP7B1,NTN1,HOMER2,HOMER1,IL27RA,SH3BP5,ROCK2,SLC25A27,STX
8,ADAMTS2,GAL3ST1,GDF15,NPEPPS,BAG3,CARTPT,TCL1B,SNCAIP,ECE2,HDA
C9,ACAP1,KEAP1,DDX46,SNAP91,SV2A,NCAPD2,HS3ST1,MED12,KCNE3,TANK,S
RA1,BCL2L11,SH2B3,IL18BP,COL4A3BP,PPIF,CTDSP2,MPHOSPH6,DDX39A,GD
F11,CALCOCO2,KCNMB2,GPHN,ABCC4,RAMP2,STUB1,LILRB2,APC2,LAMC3,C
CL26,WARS2,IRF9,SYCP2,RACK1,CPQ,RAPGEF3,PRMT5,CFDP1,COG5,ATP5PD,
SLC9A6,NXF1,PITRM1,SSSCA1,SLC35A1,NPC2,LBX1,DCTN6,EBP,USP39,NRG3,P
TGES3,CHL1,ARPP19,HSPH1,CPLX2,CPLX1,HPSE,PGRMC1,C1QL1,MMP24,PRD
X3,PRSS21,STIP1,TMED10,METAP2,DSTN,STMN2,FAF1,SLC7A9,TBC1D8,WDR45,
WDHD1,HRH3,PHB2,MGLL,NLGN1,ARHGEF15,CARD8,SBNO2,SORCS3,NCOA6,
NMNAT2,GRAMD4,RCOR1,UBXN4,ASTN2,CLUH,NUP160,WASHC4,SYNE1,UBR4,

SIRT5,GRIP1,NPTXR,KCTD2,CLCF1,SEC14L2,GTPBP4,PADI4,DDAH1,NTSR2,PRND,PPP1R15A,SLC7A11,TMEFF2,RAB38,EID1,PRPF6,FBXO7,BRI3,FBXL2,CHMP2B,LRP10,PRPF31,EPC2,FBXO2,CLDN17,HSPB8,SEZ6L2,SDCBP2,NAAA,SIT1,TNFRSF21,PDCD4,CNTN6,RND1,UBE2S,TMEM176B,SCG3,NCAPH2,HOOK2,SNX12,SLC2A8,NOP53,SLC40A1,SOCS7,IL22,GEMIN4,MBL3P,PCA3,SHANK1,ASCC1,TMED7,FIS1,CRYL1,UTP11,IRAK4,MZB1,CD320,ECSIT,NRN1,HOOK1,UBR5,BIN2,LCMT1,NAT8B,GDE1,GHRL,IL17D,LRP1B,FXYD6,CNTN5,POLE3,TREM1,PDP1,CASZ1,FOCAD,SLC25A38,TAPBPL,NUDT11,SBNO1,ADI1,FBXW7,SLC30A10,PLXNA3,ATF7IP,HHAT,LMO3,MYO5C,APOM,CFC1,KIAA1217,BTNL2,CTNNBL1,SLC2A9,ANKS1B,SPPL2B,DUSP22,PDSS2,HRASLS,CAMK1D,CD177,RTN4,AICDA,AMIGO1,KIDINS220,HECW2,SORCS2,STIM2,ALS2,LSM2,GRHL3,PTBP2,NLRC4,JAM2,NTN4,TRPV4,SRR,BCAN,NEUROD6,GFRA4,TNMD,MOAP1,NOC3L,SIL1,GREM2,GORASP1,AD6,NBEAL1,EFHD2,LIN28A,ECHDC3,PTCD2,ZC3H14,RIN3,NANOG,SCD5,PANK2,PTGES2,TET1,DNAJC5,LPAL2,SLC19A3,MAP1LC3B,NETO1,RNF146,QRFPR,HOOK3,BRSK1,MAK16,MAP1LC3A,NT5C1A,PPP1R9B,MGARP,PTPN5,ATG4C,SHANK3,WNT3A,NAV2,FATE1,ZNF628,GADD45GIP1,LMF2,SPECC1,MYOCD,CADPS2,FTMT,FOXQ1,AZIN2,DBA2,SLC26A7,GRIN3A,GRIN3B,APOA5,TPH2,FRMD6,SOCS4,OSCAR,CD200R1,LRRC15,OCIAD2,SLC32A1,CBLN4,SREK1,PHF13,IL23R,PROM2,ZDHHC15,PTCRA,DCP1B,KHDRBS2,MPEG1,KIF6,EBF3,PHYHD1,ANKK1,IFNL3,DAOAAS1,GOLGA6L2,LAMA1,ZSCAN1,SYPL2,C20orf203,SREK1IP1,RAB7B,TREML1,NANOS3,OSTN,QRFP,AMIGO2,HCN1,TICAM2,ATP9B,CHCHD10,MIRLET7D,MIR100,MIR137,MIR144,MIR155,MIR15A,MIR188,MIR195,MIR19B1,MIR20A,MIR200B,MIR214,MIR219A1,MIR22,MIR222,MIR23B,MIR26B,MIR296,MIR30E,ASS1P1,MIR339,MIR375,MIR451A,MIR485,NPS,MIR455,LOC646506,GMNC,SCFV,NCF1,GGTLC5P,FAM72B,MIR545,MIR590,MIR603,MIR616,SNORD118,TSPY3,GGTLC3,TSPY4,GGT2,CCR2,GGTLC4P,AD10,CDKN2BAS1,MIR937,AD14,AD12,TSPY10,MIR1185-1,TMED7-TICAM2,FAS-AS1,PARK16,MTRNR2L12,MIR4504,GDNF-AS1,THRA1/BTR,CST12P,LOC107987479
